# 

**Published:** 1961-01

**Authors:** 


### MEDICAL JOURNAL OF THE SOUTH-WEST

*(Incorporating the Bristol Medico-Chirurgical Journal)*

Established 1883

VOL. 76 (i) January 1961 NO. 279

### Contents

DISEASES DUE TO MEDICAL TREATMENT . . *C. Bruce Perry* 1

RESTORATIVE RESECTION OF THE RECTUM—
ITS HISTORY AND PRESENT STATUS . . *C. Patrick Sames* 7

THE GERIATRIC SERVICE. . . . *T. W. Lloyd* 12

RESEARCH ON RHEUMATIC DISEASES . . *G. D. Kersley* 20

CHRONIC OSTEOMYELITIS WITH MATURATION
ARREST OF GRANULOCYTES . *Clinical Pathological Conference* 25

NOTES AND NEWS . . . . . . . . 35

*PUBLISHED QUARTERLY*

*ANNUAL SUBSCRIPTION* 16/- *POST FREE*

PUBLISHED UNDER THE AUSPICES OF THE
BRISTOL MEDICO-CHIRURGICAL SOCIETY

### Editorial Committee

Dr. J. E. CATES, Department of Medicine, Bristol Royal Infirmary. *Editor.*

Dr. N. J. BROWN, Southmead Hospital, Bristol. *Assistant Editor.*

Dr. A. B. RAPER, Department of Pathology, Bristol Royal Infirmary. *Assistant Editor.*

Dr. J. MACRAE, Ham Green Hospital.

Dr. R. C. WOFINDEN, Central Clinic, Bristol.

Mr. A. E. S. ROBERTS, Medical Library, University of Bristol. *Secretary.*

### *Local Correspondents*

*Bath* . . Mr. A. D. BATEMAN, 3 The Circus, Bath.

*Exeter* . . Dr. L. N. JACKSON, Mount Jocelyn, Crediton, Devon.

*Gloucester* . Dr. R. F. JARRETT, 9 College Green, Gloucester.

*Plymouth* . Dr. C. R. CROFT, Lexden, Hartley Av., Plymouth.

*Taunton* . Dr. M. MCALLEY, 1 The Crescent, Taunton.

*Torquay* . Dr. A. ROBINSON THOMAS, 20 Devon Square, Newton Abbot, Devon.

*Truro* . . Dr. F. D. M. HOCKING, St. Andrews, Perranporth, Cornwall.

*Yeovil* . . Mr. J. A. VERE NICOLL, Knapp House, Yeovil, Somerset.

### NOTICE TO CONTRIBUTORS

Original articles are invited, provided they have not been published elsewhere. Notes of forthcoming events, meetings held, degrees conferred, staff appointments, and other local news are welcomed. The editorial committee reserves the right to make literary corrections.

Illustrations should always be supplied ready for reproduction. All re-touchings or other operations required will be charged to the author. Line drawings should be drawn in Indian ink. We regret that we must ask authors to pay for making blocks, if this is necessary.

J. W. ARROWSMITH LTD., WINTERSTOKE ROAD,
BRISTOL.

### *Notes and News*

### PROGRAMMES FOR 1961

### B.M.A. (Barnstaple Division)

*Hon. Secretary:* Dr. D. T. C. Barlow, 110 Boutport Street, Barnstaple

25th Jan.: **A symposium.**

22nd Feb.: **A lecture on Steroid therapy and its hazards.**

29th Mar.: **Clinical Meeting:**

26th Apr.: To be arranged.

10th May: **Annual General Meeting.**

### B.M.A. (Bristol Division)

*Hon. Secretaries:* Dr. A. S. Anderson, 10 Leigh Road, Bristol 8 and Dr. H. Temple Phillips, Berkeley House, Charlton Road, Westbury-on-Trym, Bristol

19th Apr.: 8.30 p.m. at Royal Fort. **"Accidents, the uncontrolled epidemic of this century".** By Mr. W. Gissane, CH.M., F.R.C.S.

2nd May: 8 p.m. at Ashton Country Club. Informal Reception.

24th May: (provisional date): **Clinical Meeting at Bristol General Hospital.**

16th June: (provisional date): 4.30 p.m. in the Garden Hall, Royal Fort. Reception for students sitting for final M.B., Ch.B. examinations.

Other meetings to be arranged.

### CORNWALL CLINICAL SOCIETY

*Hon. Secretaries:* Dr. B. A. Gwynne Jenkins, Central Chest Clinic, Tuckingmill, Cornwall, and Dr. H. S. Bennett, Royal Cornwall Infirmary, Truro.

13th Jan.: **Clinical Evening.** Falmouth Hospital.

2nd Feb.: **Clinical Evening.** Royal Cornwall Infirmary.

2nd Mar.: **"The Right to Die".** By Dr. A. E. Clark-Kennedy, M.D., F.R.C.P., Royal Cornwall Infirmary.

24th Mar.: B.M.A. annual lecture. **"Hypertension".** By Professor M. Rosenheim, M.D., F.R.C.P. Royal Cornwall Infirmary.

5th May: **Clinical Demonstrations.** Redruth Hospital.

1st June: Presidential Lecture and Annual General Meeting. Royal Cornwall Infirmary.

### DEVON AND EXETER MEDICO-CHIRURGICAL SOCIETY

*Hon. Secretary:* Dr. F. S. W. Brimblecombe, 6 Barnfield Crescent, Exeter

12th Jan.: at 8.15 p.m. **"Tumours of the Bladder".** Presidential address.

26th Jan.: at 3 p.m. **Clinical Meeting.** Cases available at 3 p.m., followed by tea at 4 p.m. At 4.15 p.m. **"Haematological Diagnosis".** By Dr. J. O. P. Edgecumbe. Followed by discussion of the cases.

16th Feb.: at 8.15 p.m. **"Some momentous operations".** By Mr. A. Dickson-Wright, M.S., F.R.C.S.

16th Mar.: at 8.15 p.m. **Annual General Meeting and Clinico-pathological demonstrations.**

27th Apr.: at 8.15 p.m. **Clinical Meeting** at the Princess Elizabeth Orthopaedic Hospital.

### SOUTH SOMERSET MEDICAL CLUB

*Hon. Secretary:* Dr. D. M. Barry, X-ray Department, Yeovil General Hospital

19th Jan.: **Dinner and Dance.**

16th Feb.: **"Dermatology".** By Dr. Robert Warin.

16th Mar.: Symposium, **"Deafness".** By Mr. Graeme Allan, F.R.C.S. and Mr. Peter Hugill, F.R.C.S.

20th Apr.: **"Russian Experiences".** By Dr. John Hunt. Joint meeting with College of General Practitioners.

### TORQUAY AND DISTRICT MEDICAL SOCIETY

*Hon. Secretaries:* Dr. D. K. MacTaggart, Health Department, St. Marychurch Town Hall, Torquay, and Dr. J. B. Taylor, Corner Place, Dartmouth Road, Paignton.

28th Jan.: **"How Deaf Children learn to hear".** By Miss Edith Whetnall, M.S., F.R.C.S.
23rd Feb.: **"Portal Hypertension".** By Mr. A. H. Hunt, M.A., D.M., M.CH., F.R.C.S.
17th Mar.: **"The use of Corticosteroids in Respiratory Disease".** By Dr. J. G. Scadding, M.D., F.R.C.P.
13th Apr.: **Clinical Meeting.**
11th May: **Annual General Meeting** at Victoria Hotel, Torquay.
Meetings are held at the Torbay Hospital at 8.30 p.m. unless otherwise stated.

